# A case of squamous cell carcinoma arising from a suprapubic cystostomy tract

**DOI:** 10.1186/1471-2490-11-20

**Published:** 2011-10-03

**Authors:** Hiroki Ito, Masayuki Arao, Hanako Ishigaki, Noboru Ohshima, Ayako Horita, Ikuo Saito, Kotaro Hirai

**Affiliations:** 1 Department of Urology, National Hospital Organization Sagamihara National Hospital, Kanagawa, Japan; 2 Department of Dermatology, National Hospital Organization Sagamihara National Hospital, Kanagawa, Japan; 3 Department of Pathology, National Hospital Organization Sagamihara National Hospital, Kanagawa, Japan

## Abstract

**Background:**

Patients with spinal cord injury and a chronic indwelling urinary catheter are known to have an increased risk of bladder malignancy. However, squamous cell carcinoma (SCC) of the epidermis around a suprapubic cystostomy is relatively rare. Here, we report a case of lower abdominal SCC arising from the suprapubic cystostomy tract.

**Case presentation:**

A 58-year-old man with a complete spinal cord injury was referred to our hospital with a chief complaint of an abdominal mass. Abdominal enhanced computed tomography (CT) showed a 7-cm mass surrounding the suprapubic cystostomy and bilateral inguinal and para-aortic lymph nodes metastasis. Histopathological examination of percutaneous biopsy specimens was performed. The diagnosis was stage IV (cT4N1M1) epidermal SCC, which was treated with palliative external radiation therapy.

**Conclusion:**

The SCC in this case was thought to arise from mechanical stimulus of the suprapubic cystostomy. Physicians and patients should pay careful attention to any signs of neoplasms with long-term indwelling catheters, such as skin changes around the suprapubic cystostomy site. This case presentation is only the fourth report of SCC arising from the suprapubic cystostomy tract in the literature. In cases of unresectable tumors and contraindications to chemotherapy, palliative radiotherapy may lead to disease remission and symptom relief.

## Background

Patients with spinal cord injury and a chronic indwelling urinary catheter are known to have an increased risk of bladder malignancy. This condition has been attributed to chronic inflammation and mechanical stimuli from the catheter. Chronic bladder irritation or infection is often associated with squamous metaplasia [1]. Kaufman et al. [2] reported that squamous metaplasia is more likely in patients with indwelling catheters placed for more than 10 years than in those with indwelling catheters for less than 10 years (80% vs 42%). In particular, the clinical significance of keratinizing squamous metaplasia in the setting of indwelling urinary catheters remains unclear, but some studies have linked this entity to the development of invasive squamous cell carcinoma (SCC) [3].

This can be considered in light of the fact that epidermal SCC is the second most common type of skin cancer and most cases are caused by exposure to the sun's harmful ultraviolet rays or to mechanical stimuli. We report here a case of SCC that developed in the skin around a suprapubic cystostomy.

## Case Presentation

A 58-year-old man with a complete spinal cord injury was referred to our hospital in August 2010 with a chief complaint of a severely inflamed abdominal mass. The spinal cord injury had resulted in the absence of sensation below the waist and chronic neurogenic bladder. The patient also had a history of an intracranial hemorrhage, from 4 years prior to his presentation, which had severely impaired some higher cognitive functions.

For bladder management, the patient had undergone a percutaneous cystostomy with placement of an indwelling catheter, about 35 years before his presentation. This was done due to the patient's difficulty performing clean, intermittent catheterization on a regular basis. The suprapubic cystostomy catheter was changed once a month at another urology clinic.

**Figure 1:**
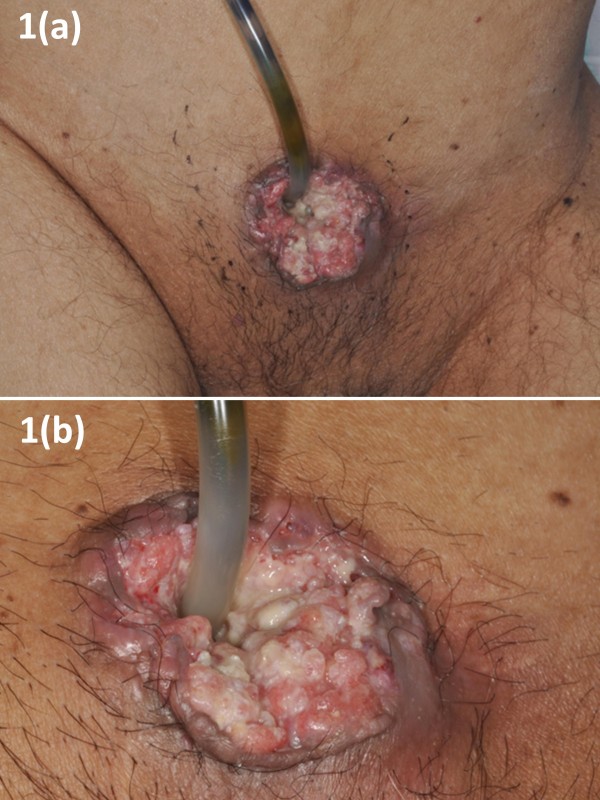
**Abdominal Mass surrounding a suprapubic cystostomy**. (a) The mass(50 mm diameter) was noted around the suprapubic catheter. (b) Closer view of the abdominal mass surrounding a suprapubic cystostomy.

**Figure 2:**
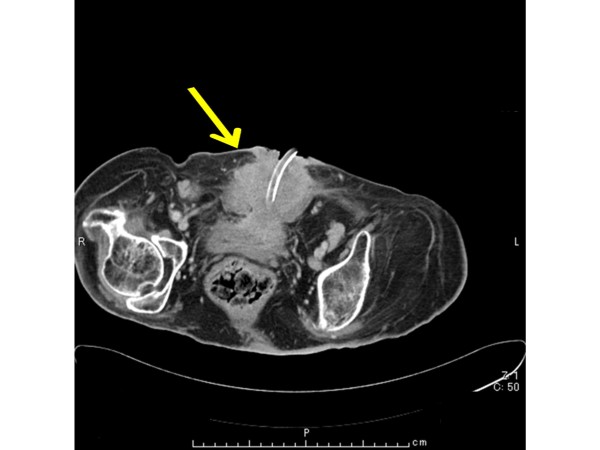
**An enhanced abdominal CT showed a mass (72 mm × 63 mm) surrounding suprapubic**.

The physical examination revealed an abdominal mass surrounding a suprapubic cystostomy (Figure 1a). The skin around the mass was erythematous, edematous, and a foul-smelling, purulent discharge was present (Figure 1b). Blood analysis revealed the following abnormal values: albumin 2.3 g/dl, hemoglobin 8.6 g/dl, elevated white blood cells to 11,200/μl, and C-reactive protein 11.89 mg/dl. Urinalysis revealed leukocytes (< 100/HPF) and hematuria (50-99/HPF). Urine cytologic evaluation was class α and atypical squamous cells were seen on microscopic examination. Enhanced chest and abdominal CT (Figure 2) showed a mass (72 mm × 63 mm) surrounding the suprapubic cystostomy and enlarged bilateral inguinal and para-aortic lymph nodes. Chest and anterior mediastinal lesions showed no specific findings. A cystoscopy could not be performed because the patient had a lower-extremity contracture deformity.

**Figure 3:**
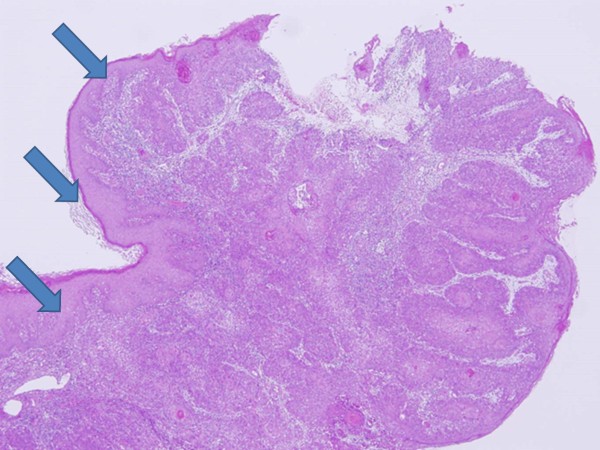
**Microscopic findings (hematoxylin and eosin stain): Well differentiated squamous cell carcinoma were found**. Front formation(arrow head) were observed at the border between carcinoma cells and normal epithelial cells subcutaneously.

After the local inflammation of the abdominal mass was resolved with intravenous antibiotics, a percutaneous biopsy was performed under local anesthesia. The histopathological examination of the biopsy specimens from the tumor suggested SCC (Figure 3). Thus, stage IV (cT4N1M1) epidermal SCC was diagnosed and subsequently treated with palliative external radiation therapy. A dose of 56 Gy was administered over 5 weeks to the pelvic area including the primary tumor and inguinal metastatic lymph nodes. The primary tumor and metastatic lymph nodes responded partially to this therapy. The period after radiotherapy was uneventful. The patient has remained asymptomatic during the subsequent 6 months.

## Discussion

The most common bladder tumors in patients with spinal cord injury are SCC (33-46.9%), urothelial carcinoma (31.3-55%), and adenocarcinoma (9.4-10%) [4-6]. In the literature SCC is more common in patients with indwelling urethral and suprapubic catheters than other forms of bladder management. The incidence of SCC of the bladder in patients with indwelling catheters for more than 10 years is 10% [7]. In a study of 48 patients, the mean time between spinal cord injury and the first bladder malignancy diagnosis was 22.6 years [8].

In the present case, we were unable to perform a cystoscopy and assess the bladder mucosa for a possible origin of the tumor. However, we concluded that the SCC developed not from the bladder but from the epidermis around the suprapubic catheter. This conclusion was based on the absence of gross hematuria during follow-up, and a class II urine cytologic evaluation. In addition, front formation [9] was observed subcutaneously at an obvious border between normal epithelial cells and carcinoma cells (Figure 3 arrow). These findings indicated the origin of SCC was squamous epithelial cells. To our knowledge, the present case is only the fourth report of SCC arising from the suprapubic cystostomy tract in the literatures [7,10,11].

We believe that this case of SCC was caused by chronic exposure of the cystostomy site to the mechanical stimuli from the indwelling suprapubic catheter. However, the risk of SCC would not have been eliminated by intermittent catheterization, as demonstrated by reported cases bladder malignancies in patients who perform intermittent catheterization [4,6,8].

The only acceptable treatment for deeply invasive but localized SCC arising from a suprapubic cystostomy tract is radical cystectomy and urinary diversion [11]. In this case, the patient's tumor was not localized and had metastasized to the inguinal and para-aortic lymph nodes. In such cases, chemotherapy is considered, but was contraindicated in this patient due to his poor performance status. Thus, the patient underwent external radiation therapy as a palliative treatment. This treatment led to partial disease remission and good palliation of symptoms and it would appear that palliative radiation therapy (a total of 56 Gy) has a role to play in the palliation of metastatic SCC, with good relief of symptoms.

We have presented here a rare case of epidermal SCC in a patient with a suprapubic cystostomy. Physicians and patients should pay close attention to any suspicious signs associated with such long-term cystostomy sites, including skin changes. In this case, other urologists who were changing the catheter once a month had noticed the abdominal mass for 6 months before referring the patient to our clinic admission, but they had considered the cause to be hyperplasia due to benign granulation. The slow growth of the mass may have made early diagnosis difficult. This case clearly demonstrates that chronic indwelling catheters may cause malignancy of not only the bladder but also the epidermis. Thus, early detection and treatment of SCC arising from a suprapubic cystostomy tract are crucial.

## Conclusions

This case presentation is only the fourth report of SCC arising from the suprapubic cystostomy tract in the literature. In this case of unresectable metastatic SCC, palliative external radiation therapy led to partial disease remission and good relief of symptoms. It is crucial to pay attention to any suspicious signs, including skin changes around a suprapubic cystostomy, especially in the presence of a long-term indwelling catheter.

## Consent

Written informed consent was obtained from the patient for publication of this case report and any accompanying images. A copy of the written consent is available for review from the Editor-in-Chief of the journal.

## Competing interests

The authors declare that they have no competing interests.

## Authors' contributions

HI cared for the patient and drafted the report. MA, HI, NO, and KH cared for the patient and approved the final version of the manuscript. AH and IS performed histopathological examinations.

All authors reviewed the report and approved the final version of the manuscript.

## Pre-publication history

The pre-publication history for this paper can be accessed here:

http://www.biomedcentral.com/1471-2490/11/20/prepub
